# Supplementary material for: Liquid Madelung energy accounts for the huge potential shift in electrochemical systems
Source: Nat Commun. 2024 Feb 19;15:1319. doi: 10.1038/s41467-023-44582-4 (PMC10876980; doi:10.1038/s41467-023-44582-4)
Supplement: Supplementary file 1 — Supplementary Information [file 41467_2023_44582_MOESM1_ESM.pdf]

Supplementary Information

Liquid Madelung energy accounts for the huge potential shift in electrochemical systems

Norio Takenaka[1], Seongjae Ko[1], Atsushi Kitada[1], Atsuo Yamada[1,2]*

1. Department of Chemical System Engineering, School of Engineering, The University of Tokyo, Hongo 7-3-1, Bunkyo-ku, Tokyo 113-8656, Japan
2. Sungkyunkwan University Institute of Energy Science & Technology (SIEST), Sungkyunkwan University, Suwon 16419, Korea

S1. Liquid Madelung potential calculation

In this study, E_{LM} is obtained numerically by calculating the Coulombic interaction energies between the target Li^+ ion and other solvents/ions during the MD simulations under periodic boundary conditions and subsequently averaging these values over time for all Li^+ ions as follows:

$$E_{\mathrm{LM}} = \frac{1}{N_{\mathrm{Li+}}} \langle \sum_i \left(E^{tot} - E_i^{\mathrm{extracted}} - E^{\mathrm{Li+}} \right) \rangle \quad [\mathrm{S1}]$$

where E^{tot} is the total Coulombic energy of the system, $E_i^{\mathrm{extracted}}$ is the Coulombic energy of the system with the i-th Li^+ extracted, $E^{\mathrm{Li+}}$ is the Coulombic energy of the system with one Li^+, and $N_{\mathrm{Li+}}$ is the number of Li^+. Note that the Coulombic energies of the charge excess systems ($E^{\mathrm{extracted}}$ and $E^{\mathrm{Li+}}$) under periodic boundary conditions include the numerical errors due to the background charges (BC) to neutralize the system, depending on the simulation cell size. To mitigate this unavoidable effect, the simulation cell size was kept almost the same for each electrolyte. The errors are indeed negligible, because the calculated potential shifts with BC showed almost identical values with those obtained by directly summing the Coulombic interactions (without BC) among Li^+ and other solvents/ions up to the electrostatically shielded distance (i.e., 30 Å) (Fig. S11).

S2. Theoretical basis

Eq. [1] in the main text is converted as follows:

$$\Delta E_{\mathrm{Li/Li+}} = \frac{\Delta \mu_{\mathrm{Li+}}}{F} \quad \text{[S2]}$$

where $\mu_{\mathrm{Li+}}$ is the chemical potential (or partial molar Gibbs energy) of Li^+. Here, the chemical potential is rewritten using the partial molar enthalpy and partial molar entropy, and the enthalpy term can be described as the sum of E_{LM} for Li^+ and the other terms:

$$\mu_{\mathrm{Li+}} = H - TS = (E_{\mathrm{LM}} + E_{\mathrm{other}}) - TS. \quad \text{[S3]}$$

Therefore, the difference in the chemical potential is given by

$$\Delta\mu_{\mathrm{Li+}} = (\Delta E_{\mathrm{LM}} + \Delta E_{\mathrm{other}}) - T\Delta S. \quad \text{[S4]}$$

If $\Delta E_{\mathrm{LM}} >> \Delta E_{\mathrm{other}} - T\Delta S$, or ΔE_{LM} is the predominant contribution to the enthalpy term and the entropy term is relatively small, $\Delta E_{\mathrm{Li/Li+}}$ can be expressed as follows:

$$\Delta E_{\mathrm{Li/Li+}} = \frac{\Delta\mu_{\mathrm{Li+}}}{F} \cong \frac{\Delta E_{\mathrm{LM}}}{F}. \quad \text{[S5]}$$

In Figure 3a–c, ΔE_{LM} is expressed in the units of electron volt (eV), obtained by dividing the ΔE_{LM} in Joule (J) units by the Faraday constant F. Thus, eq. [S5] explains why the experimental values of $\Delta E_{\mathrm{Li/Li+}}$ are reproduced well by ΔE_{LM}. According to eq. [1] in the main text and eq. [S5], ΔE_{LM} varies logarithmically, as shown in Figure 3a–c (red lines). However, the entropy term and bonding effect are not considered in the present simple model, requiring further sophistication in modeling to achieve higher accuracy.[1,2]

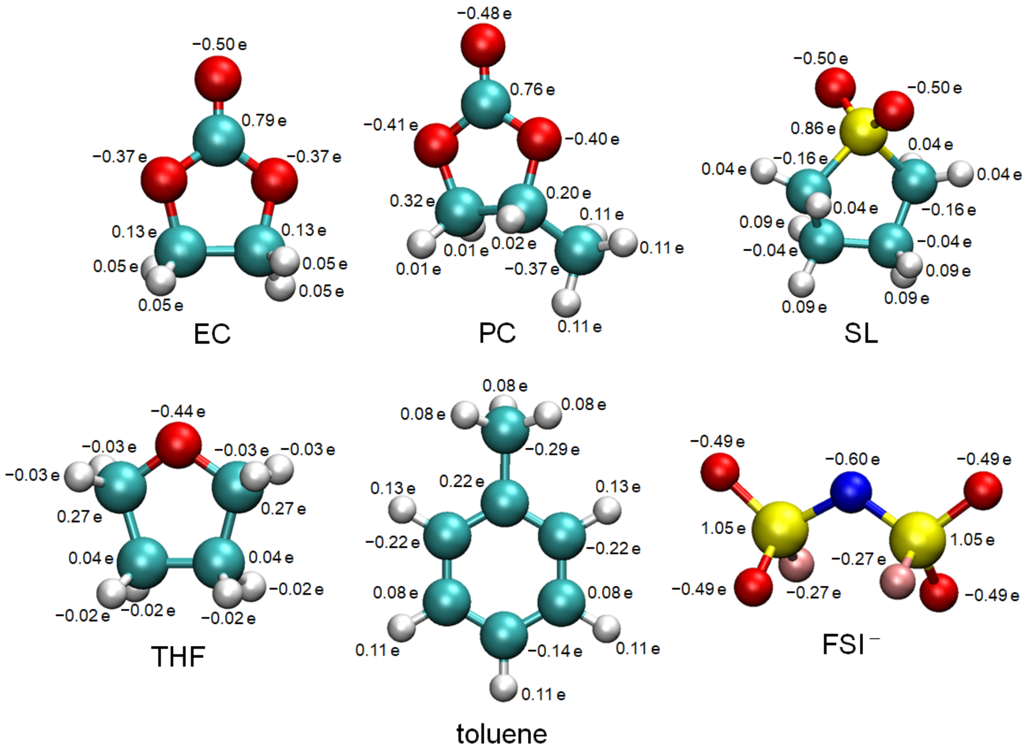

Fig. S1: Partial atomic charges of ethylene carbonate (EC), propylene carbonate (PC), sulfolane (SL), tetrahydrofuran (THF), toluene, and bis(fluorosulfonyl)imide anion (FSI^-) in MD simulations (carbon, oxygen, hydrogen, sulphur, nitrogen and fluorine are shown in cyan, red, white, yellow, blue, and pink, respectively).

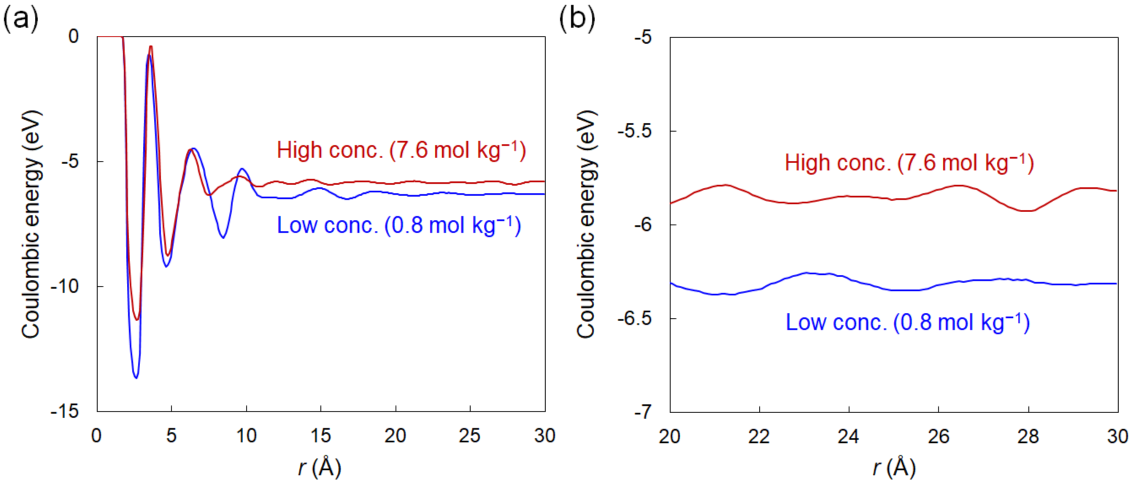

Fig. S2: (a) Coulombic energy as a function of r (distance from Li^{+}) for the 0.8 mol kg^{-1} LiFSI/EC and 7.6 mol kg^{-1} LiFSI/EC electrolytes, and (b) magnified view of the plots for $r > 20$ Å.

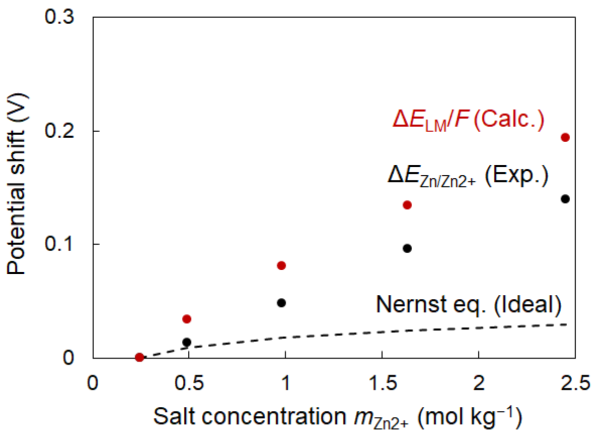

Fig. S3: Experimental potential shifts ($\Delta E_{Zn/Zn2+}$ (Exp.)) and the calculated ones from liquid Madelung potential ($\Delta E_{LM}/F$) in $Zn(TFSI)_2$/PC electrolytes as a function of salt concentration m_{Zn2+} with reference to 0.25 mol kg^{-1}. The black dashed line represents the potential shift based on the ideal Nernst equation, where γ_{zn2+} is assumed to be unity.

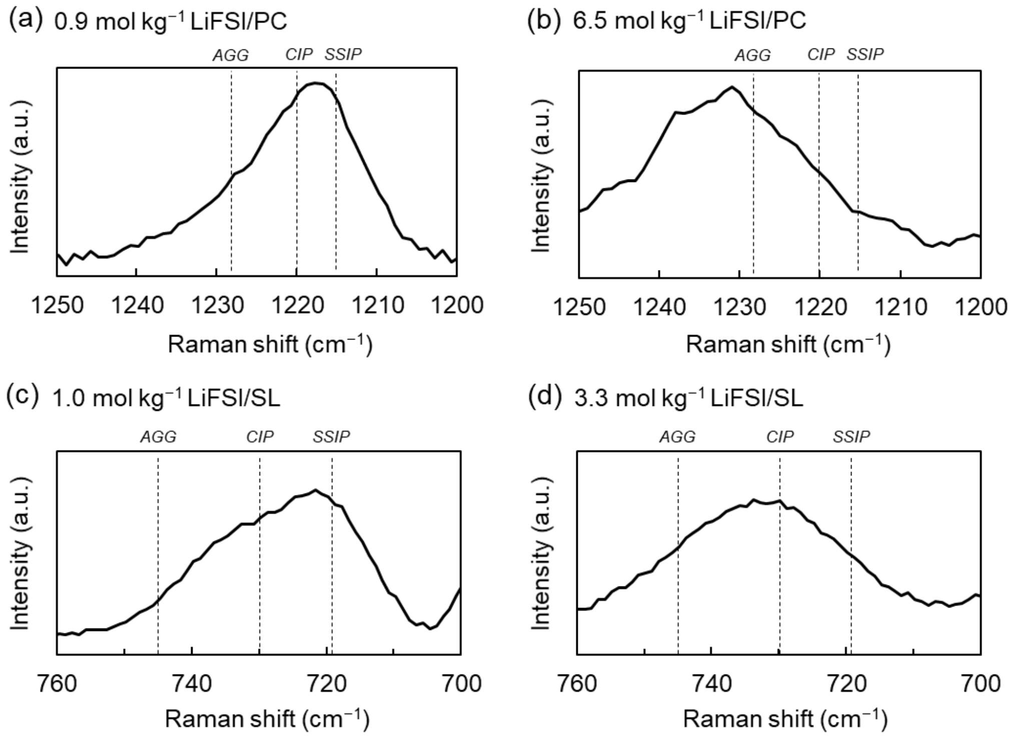

Fig. S4: Raman spectra of (a) 0.9 mol kg^{-1} LiFSI/PC, (b) 6.5 mol kg^{-1} LiFSI/PC, (c) 1.0 mol kg^{-1} LiFSI/SL, and (d) 3.3 mol kg^{-1} LiFSI/SL electrolytes. Black dashed lines show the representative position for the different coordination states of the Li^+ and FSI^- ion pairs described in the main manuscript.

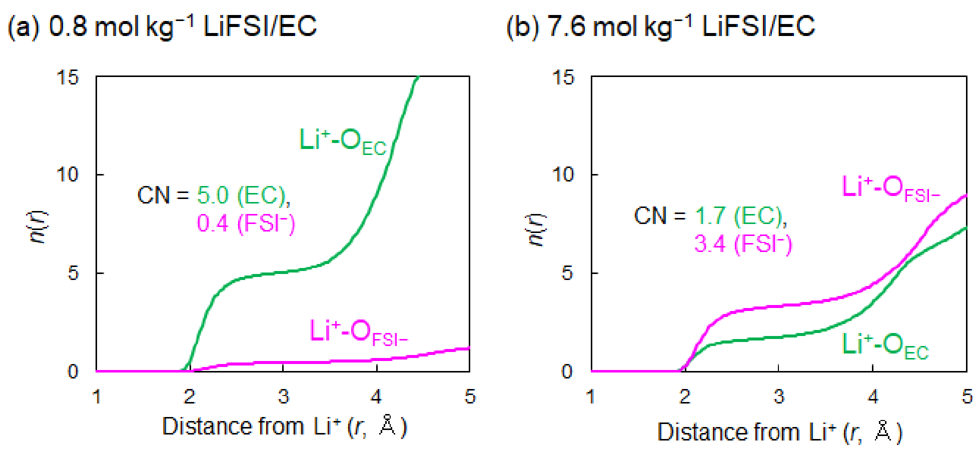

Fig. S5: Number distribution functions $n(r)$ with the coordination numbers (CN) of the first coordination sphere ($r < 2.9$ Å (green) or 3.1 Å (magenta)) in the (a) 0.8 mol kg^{-1} and (b) 7.6 mol kg^{-1} LiFSI/EC electrolytes.

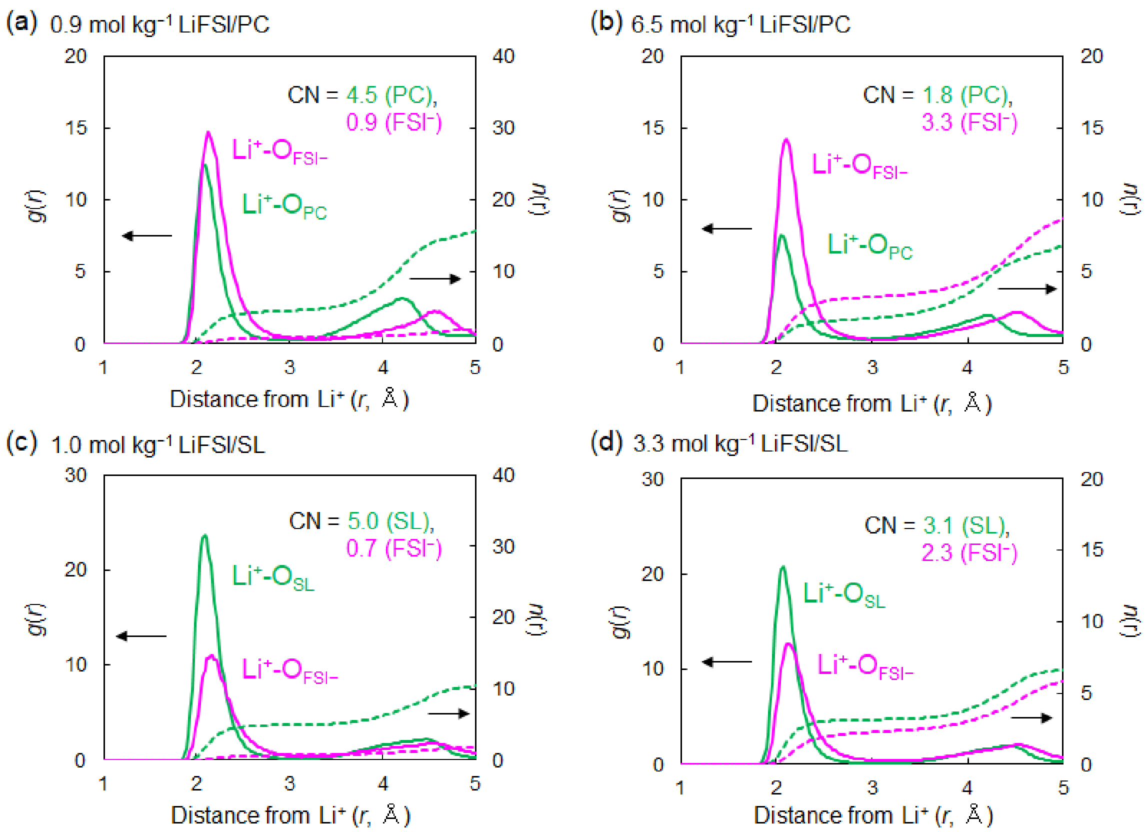

Fig. S6: Radial distribution functions $g(r)$ (solid lines) and number distribution functions $n(r)$ (dashed lines) in the (a) 0.9 mol kg^{-1} LiFSI/PC, (b) 6.5 mol kg^{-1} LiFSI/PC, (c) 1.0 mol kg^{-1} LiFSI/SL, and (d) 3.3 mol kg^{-1} LiFSI/SL electrolytes. The CNs in the first coordination sphere ($r < 2.9$ Å (green) or 3.1 Å (magenta)) are also shown.

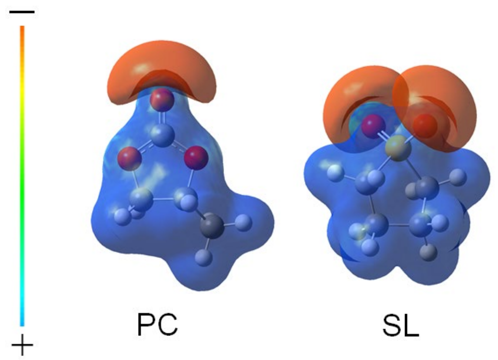

Fig. S7: Electron density isosurface mapped with molecular electrostatic potential of the PC and SL solvents (carbon, oxygen, hydrogen and sulphur shown in grey, red, white, and yellow, respectively).

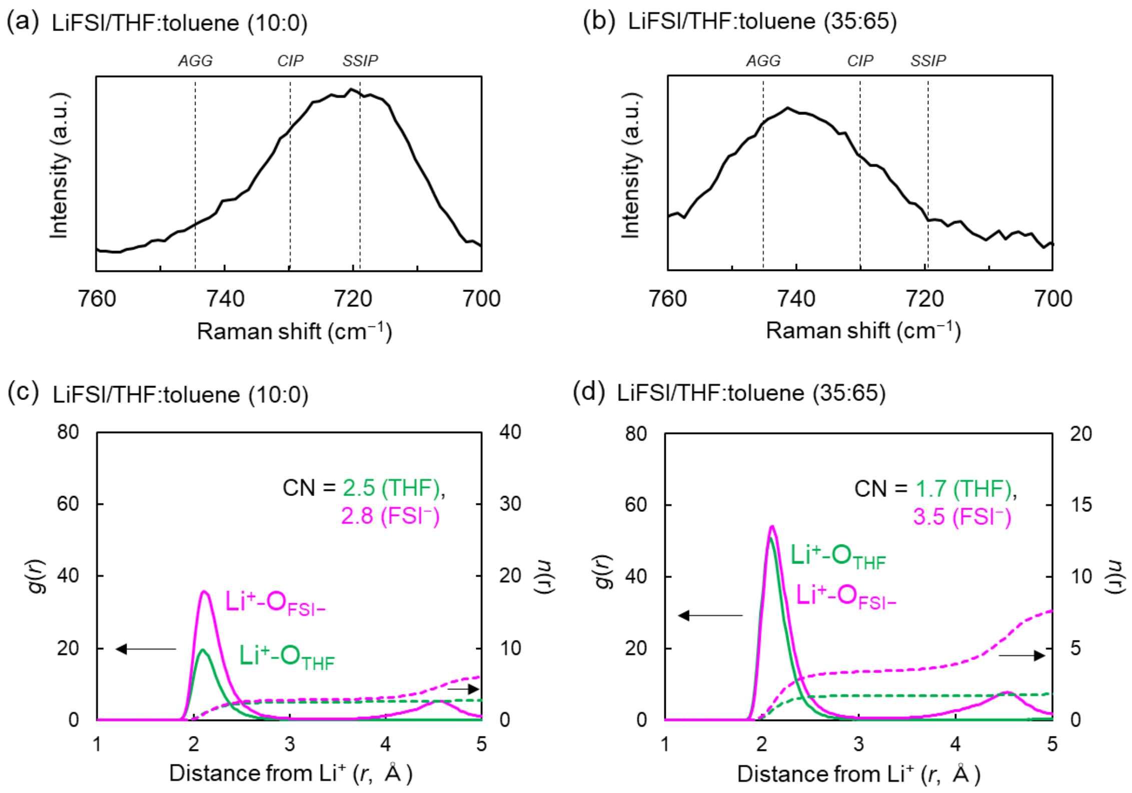

Fig. S8: Raman spectra of the (a) 1.5 mol dm^{-3} LiFSI/THF:toluene (10:0) and (b) 1.5 mol dm^{-3} LiFSI/THF:toluene (35:65) electrolytes. Black dashed lines show the representative position for the coordination state of the Li^+ and FSI^- ion pairs. Radial distribution functions $g(r)$ (solid lines) and number distribution functions $n(r)$ (dashed lines) for the (c) 1.5 mol dm^{-3} LiFSI/THF:toluene (10:0) and (d) 1.5 mol dm^{-3} LiFSI/THF:toluene (35:65) electrolytes. The CNs in the first coordination sphere (r < 3.1 Å (green) or 3.3 Å (magenta)) are also shown.

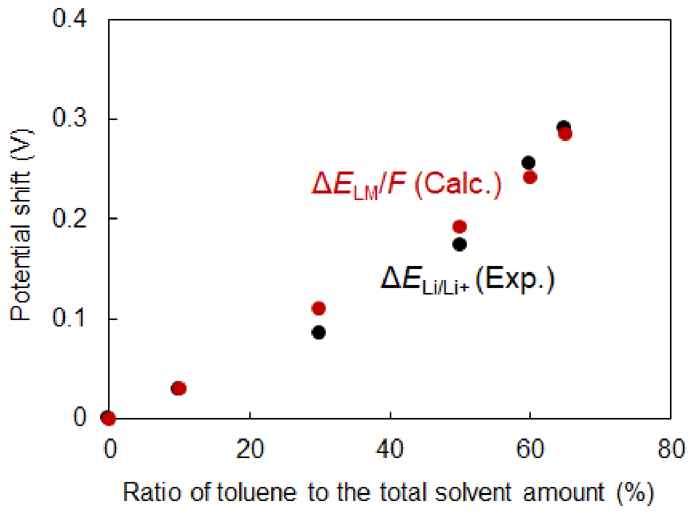

Fig. S9: Experimental data for $\Delta E_{\mathrm{Li/Li+}}$ and the calculated potential shifts of liquid Madelung potential ($\Delta E_{\mathrm{LM}}/F$) defined as the shift from the data for 1.5 mol dm^{-3} LiFSI/THF electrolyte without toluene and plotted as a function of the ratio of toluene to the total solvent amount (%).

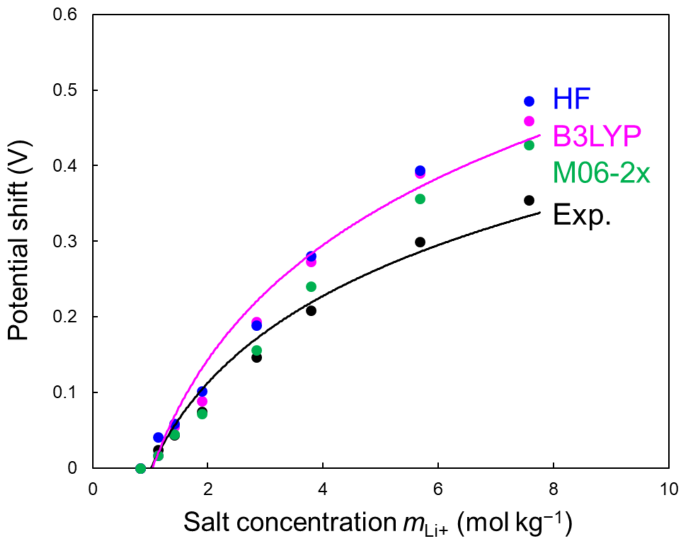

Fig. S10: Experimental data for $\Delta E_{Li/Li+}$ (black circles) and calculated potential shifts of liquid Madelung potentials ($\Delta E_{LM}/F$) using RESP charges at various calculation levels (magenta circles: B3LYP/cc-pvdz (this work), blue circles: HF/cc-pvdz, green circles: M06-2x/cc-pvdz) in LiFSI/EC electrolytes as a function of salt concentration m_{Li+} with reference to 0.8 mol kg^{-1}.

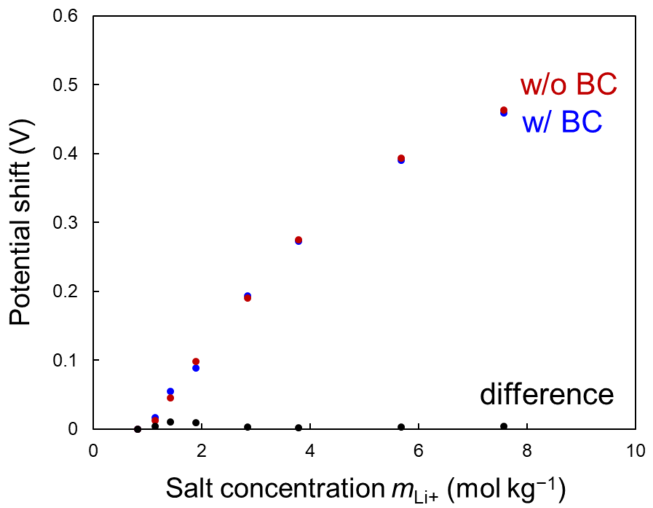

Fig. S11: Calculated potential shifts of liquid Madelung potentials ($\Delta E_{LM}/F$) using two different methods (blue circles: with background charges (BC) (this work), red circles: without BC) in LiFSI/EC electrolytes as a function of salt concentration m_{Li+} with reference to 0.8 mol kg^{-1}. Differences with and without BC are also indicated by black circles. The potential shifts without BC are obtained by directly summing the Coulomb interactions among Li^+ and other solvents/ions up to the electrostatically shielded distance (i.e., 30 Å).

Table S1: Molarities, simulation cell lengths, and numbers of molecules/ions for MD simulations of the LiFSI/EC, LiFSI/PC, and LiFSI/SL electrolytes at various concentrations.

Electrolyte (Salt-to-solvent molar ratio, *n*:*n*)	Molarity (mol kg^{-1})	Cell length (Å)	*N* (Li^+)	*N* (FSI^-)	*N* (Solvent)
LiFSI/EC (1:13.8)	0.8	69.2	200	200	2760
LiFSI/EC (1:10)	1.1	70.3	280	280	2800
LiFSI/EC (1:8)	1.4	68.8	320	320	2560
LiFSI/EC (1:6)	1.9	70.6	440	440	2640
LiFSI/EC (1:4)	2.8	70.3	600	600	2400
LiFSI/EC (1:3)	3.8	69.7	720	720	2160
LiFSI/EC (1:2)	5.7	71.3	1000	1000	2000
LiFSI/EC (1:1.5)	7.6	71.8	1200	1200	1800
LiFSI/PC (1:10.6)	0.9	67.9	200	200	2120
LiFSI/PC (1:8)	1.2	67.0	240	240	1920
LiFSI/PC (1:6)	1.6	67.8	320	320	1920
LiFSI/PC (1:4)	2.5	69.4	480	480	1920
LiFSI/PC (1:3)	3.3	69.4	600	600	1800
LiFSI/PC (1:2)	4.9	70.7	840	840	1680
LiFSI/PC (1:1.5)	6.5	71.5	1040	1040	1560
LiFSI/SL (1:9.5)	0.9	69.8	208	208	1976
LiFSI/SL (1:8)	1.0	69.5	240	240	1920
LiFSI/SL (1:6)	1.4	70.2	320	320	1920
LiFSI/SL (1:4)	2.1	71.8	480	480	1920
LiFSI/SL (1:3)	2.8	71.8	600	600	1800
LiFSI/SL (1:2.5)	3.3	72.9	720	720	1800

Table S2: Simulation cell lengths and numbers of molecules/ions for MD simulations of 1.5 mol dm^{-3} LiFSI/THF:toluene electrolytes with various THF-to-toluene molar ratios (*n*:*n*).

Electrolyte (THF-to-toluene molar ratio, *n*:*n*)	Cell length (Å)	*N* (Li^+)	*N* (FSI^-)	*N* (THF)	*N* (toluene)
1.5 M LiFSI/THF (10:0)	70.2	320	320	2272	0
1.5 M LiFSI/THF:toluene (9:1)	70.8	320	320	2016	224
1.5 M LiFSI/THF:toluene (7:3)	72.3	320	320	1587	680
1.5 M LiFSI/THF:toluene (5:5)	73.7	320	320	1136	1136
1.5 M LiFSI/THF:toluene (4:6)	74.4	320	320	912	1368
1.5 M LiFSI/THF:toluene (35:65)	74.7	320	320	792	1470

Supplementary References

1. Dokko, K., Watanabe, D., Ugata, Y., Thomas, M. L., Tsuzuki, S., Shinoda, W., Hashimoto, K., Ueno, K., Umebayashi, Y. & Watanabe, M. Direct evidence for Li ion hopping conduction in highly concentrated sulfolane-based liquid electrolytes. *J. Phys. Chem. B* **122**, 10736-10745 https://doi.org/10.1021/acs.jpcb.8b09439 (2018).
2. Xu, J. High-entropy electrolytes in boosting battery performance. *Mater. Futures* **2**, 047501 10.1088/2752-5724/ace8ab (2023).
